# A Randomized Study of a Comfort-Oriented Daytime Double-J Stent Removal Protocol: Effects on Pain, Anxiety, and Patient Satisfaction

**DOI:** 10.5152/tud.2026.25137

**Published:** 2026-06-11

**Authors:** Xiaoyan Xu, Shuo Wu, Qingqing Yu, Rong Tang

**Affiliations:** 1 Department of Urology, The First People’s Hospital of Lin’An District, Hangzhou, China; 2 Department of Anesthesiology, The First People's Hospital of Lin’An District, Hangzhou, China

**Keywords:** Anxiety, comfort-oriented medical care, daytime surgery, double-J stent, stent removal

## Abstract

**Objective:**

To evaluate the clinical impact of a modified daytime double-J (DJ) stent removal model based on comfort-oriented medical care on patient pain, anxiety, procedural efficiency, and satisfaction.

**Methods:**

Between January 2024 and June 2025, 160 patients undergoing ureteroscopy or percutaneous nephrolithotomy requiring DJ stent removal were randomly assigned to a comfort group (n = 80) or a conventional group (n = 80). The comfort group received structured preoperative education, intravenous sedation combined with urethral local anesthesia, pressurized irrigation-assisted cystoscopic removal, and postoperative follow-up. The conventional group underwent traditional outpatient cystoscopic stent removal. Outcomes included Visual Analog Scale (VAS) pain scores, Self-Rating Anxiety Scale (SAS) scores, stent removal time, patient satisfaction, and postoperative complications.

**Results:**

The comfort group reported significantly lower 24-hour VAS pain scores than the conventional group (2.6 ± 0.9 vs. 5.5 ± 1.0, *P *< .001). Baseline SAS scores were significantly lower in the comfort group than in the conventional group (30.2 ± 5.7 vs. 46.2 ± 7.0, *P *< .001), and postoperative SAS scores were 28.4 ± 4.7 vs. 42.0 ± 6.8, both *P *< .001. Stent removal time was shorter in the comfort group (1.90 ± 0.35 minutes vs. 2.20 ± 0.50 minutes, *P *= .014). Satisfaction scores and satisfaction rates were higher in the comfort group (4.6 ± 0.5 vs. 3.6 ± 0.7, 96.2% vs. 70.0%, *P *< .01). The 24-hour postoperative complication rate was low and not statistically significant (2.5% vs. 2.5%, *P *> .05).

**Conclusion:**

The comfort-oriented daytime stent removal model significantly reduces procedural pain and anxiety while improving patient satisfaction without increasing short-term complications.

**Main Points**

- A novel multimodal protocol integrating education, sedation, and pressurized irrigation reduces stent removal pain by over 50% and significantly lowers anxiety.
- The comfort-oriented model shortens procedural time and increases patient satisfaction rates to 96.2% compared to 70.0% in conventional care.
- Addressing both physiological and psychological stressors offers a safe, feasible framework for standardizing patient-centered care in outpatient urology.

## Introduction

Ureteral stenting is widely used following urolithiasis surgeries such as ureteroscopy (URS) or percutaneous nephrolithotomy (PCNL) to maintain urinary drainage, prevent obstruction, and facilitate healing of the ureteral mucosa.^1^^,^^2^ However, removal of double-J (DJ) ureteral stents remains a source of considerable discomfort for many patients.^3^ The procedure, typically performed with a rigid or flexible cystoscope, often induces urethral pain, lower abdominal discomfort, dysuria, and anxiety, all of which can negatively impact patient satisfaction and compliance.^4^

Traditional stent removal approaches prioritize procedural efficiency and technical success but often neglect the patient’s psychological and physiological comfort. Several studies have reported that up to 60% of patients experience moderate-to-severe pain during stent removal, with anxiety levels frequently elevated due to fear of the unknown or anticipated pain.^5^^,^^6^ These adverse experiences can result in increased procedural time, patient distress, and reduced adherence to follow-up care, underscoring the need for patient-centered interventions.

Comfort-oriented or “enhanced patient experience” approaches have been successfully applied in other urological procedures. Interventions such as preoperative counseling, distraction techniques, local anesthesia, and intravenous sedation have been shown to decrease both procedural pain and anxiety while improving patient satisfaction.^7^^-^^9^ Nevertheless, most studies focus on single interventions rather than comprehensive, workflow-integrated strategies. There remains limited literature evaluating holistic comfort-oriented models that encompass preoperative education, intraoperative optimization, and postoperative follow-up for ureteral stent removal in day surgery settings.

Given the increasing demand for efficient outpatient urological procedures and the emphasis on patient-centered care, there is a clear need to develop and evaluate systematic interventions that minimize pain and anxiety, optimize procedural efficiency, and enhance overall patient experience. In this context, a comfort-oriented stent removal protocol was designed, integrating preoperative education, intravenous sedation combined with local anesthesia, irrigation-assisted cystoscopic removal, and structured postoperative follow-up. The present study aimed to evaluate the effectiveness of this approach on pain, anxiety, procedural efficiency, patient satisfaction, and safety in a randomized cohort of patients undergoing DJ stent removal following URS or PCNL. By addressing both psychological and physiological aspects of the stent removal process, this study provides a model for implementing comprehensive comfort-oriented care in urology, with potential implications for standardizing best practices in outpatient and day-surgery settings.^10^

## Material and Methods

### Study Design and Participants

**Figure 1.**
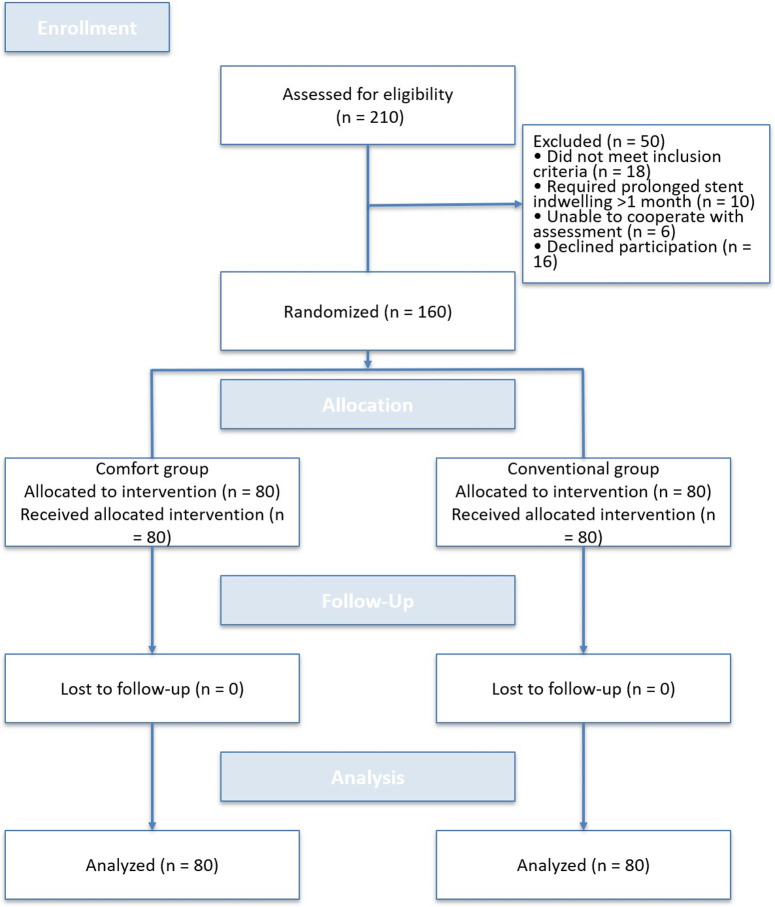
Flow diagram of the enrollment, allocation, follow-up, and analysis of the participants.

This prospective, single-center, randomized controlled trial was conducted between January 2024 and June 2025 at the Department of Urology, The First People's Hospital of Lin'an District. A total of 160 patients who underwent URS or PCNL with subsequent DJ ureteral stent placement were enrolled. After confirming eligibility and obtaining written informed consent, participants were randomly allocated in a 1:1 ratio to either the comfort (n = 80) or conventional (n = 80) group. Randomization was performed using a computer-generated sequence by an independent statistician, with allocation concealment achieved via sequentially numbered, sealed opaque envelopes (Figure 1). Ethical approval for this study was granted by the Ethics Committee of The First People's Hospital of Lin'an District (Approval No.: IRB 2023-028; Date: December 9, 2023). All procedures were conducted in accordance with the Declaration of Helsinki and relevant national guidelines.

Inclusion criteria were: age 18-75 years; ability to cooperate with the procedure and assessment; scheduled for DJ stent removal. Exclusion criteria included: any diagnosed mental or cognitive disorder; known severe urethral stricture or ongoing gross hematuria; conversion to inpatient care or occurrence of severe complications on the day of stent removal; patients requiring ureteral stent indwelling for longer than 1 month (e.g., due to ureteral stricture or recurrent hydronephrosis). Baseline characteristics—including age, sex, body mass index (BMI), surgical modality, stent indwelling duration, and history of urinary tract infection (UTI)—were comparable between groups (*P* > .05).

### Interventions

#### Conventional Group:

Participants in the conventional arm received the institution's standard protocol for DJ stent removal. The procedure was conducted in the day-surgery cystoscopy suite using a 19.8 Fr rigid cystoscope (Storz®). Two minutes prior to insertion, 10 mL of 1% lidocaine was administered at the external urethral meatus for local anesthesia. A urology attending physician then performed the stent removal. After the procedure, patients were monitored in the recovery area for approximately 30 minutes and subsequently discharged. Follow-up calls were made by research assistants on the same day and the following day to record pain scores (Visual Analog Scale (VAS)), anxiety status, and satisfaction. All steps strictly followed the urology department’s routine standard of care, with no additional preoperative education, intraoperative sedation, or pressurized irrigation assistance applied.

#### Comfort Group:

### Outcome Measures

The following key outcome measures were predefined and prospectively collected:

Pain: Pain intensity was assessed using the 10-point VAS (0 = no pain, 10 = worst imaginable pain).^14^ The VAS scores were recorded at baseline (prior to stent removal), immediately post-procedure, and 24 hours post-procedure. The primary pain endpoint was defined as the 24 hour post-procedure VAS.

Anxiety: Pre-procedural and post-procedural anxiety status was measured using the Zung Self-Rating Anxiety Scale (SAS).^15^ This validated instrument comprises 20 items scored 1-4, yielding a raw total score range of 20-80, which is then converted to an “Anxiety Index” (20-44: normal; 45-59: mild-moderate anxiety; ≥60: severe anxiety). Baseline anxiety was assessed before any educational or relaxation intervention. A predefined secondary analysis examined the difference between post- and pre-SAS scores.

Procedural Efficiency: Procedural efficiency was quantified by 2 metrics: (a) stent removal time (defined as the interval from cystoscope insertion to complete withdrawal of the stent in minutes) and (b) total outpatient time (from patient admission to discharge from the day-surgery unit, in minutes). These measures were logged in real time by a dedicated research assistant and serve as proxies for workflow impact and resource utilization.

Patient Satisfaction: Patient satisfaction with the stent removal procedure was assessed using a 5-point Likert scale, ranging from 1 = very dissatisfied to 5 = very satisfied. A score of ≥4 was prespecified as “satisfied.” This scale was developed specifically for use in this study and has not been formally validated in external populations; its use and limitations are addressed in the Discussion. Follow-up at 24 hours included an open-ended question capturing patients’ overall experience.

Complications: Complications were defined a priori to include any instance of macroscopic hematuria lasting more than 2 hours post-procedure, UTI, emergency department presentation, or hospital admission within 24 hours following stent removal. All adverse events were classified and graded according to the Clavien–Dindo classification and reported according to CONSORT harms guidelines.^16^

This study used generative artificial intelligence tools for language editing and grammar improvement during the preparation of the manuscript. However, all scientific content, interpretation of results, and final manuscript revisions were reviewed and approved by the authors.

### Statistical Analysis

Sample size estimation was based on detecting a clinically meaningful difference of 1.5 points in VAS pain scores between groups, assuming a standard deviation of 2.5, a two-sided *α* of 0.05, and 80% statistical power. The required sample size was 72 patients per group. To account for potential dropouts, 80 patients were enrolled in each group. Data were analyzed using IBM SPSS Statistics v24.0 (IBM Corp., Armonk, NY, USA). Continuous variables are presented as mean ± standard deviation (SD) and compared using independent-samples *t*-test (if normally distributed) or Mann–Whitney *U* test (if not). Categorical variables are expressed as n (%) and compared via chi-square or Fisher’s exact test as appropriate. A two-sided *P* value <.05 was considered statistically significant.

## Results

### Baseline Characteristics

A total of 160 patients were enrolled and randomized, with 80 allocated to each group. All patients completed the study without protocol violations requiring exclusion. Baseline characteristics were well balanced between groups (Table 1). Mean age was 45.0 ± 12.4 years in the comfort group vs. 46.4 ± 11.6 years in the conventional group (*P *= .462). Gender distribution (male/female: 48/32 vs. 46/34), BMI (24.3 ± 3.5 vs. 24.7 ± 3.8 kg/m^2^), and surgical type (URS/PCNL: 68/12 vs. 71/9) showed no significant differences. Stent indwelling duration was comparable (10.4 ± 2.7 vs. 10.1 ± 2.9 days, *P *= .424), reflecting institutional practice of early stent removal within 10 days for uncomplicated cases. Prior history of UTI was documented in 14 patients (17.5%) in the comfort group and 9 patients (11.2%) in the conventional group (*P *= .367).

### Pain Assessment

Pain intensity measured by VAS differed significantly between groups at both assessment points (Table 2). Immediately following stent removal, the comfort group reported lower VAS scores than the conventional group (1.8 ± 0.8 vs. 4.2 ± 1.1, *P *< .001). At 24 hours post-procedure, this difference persisted (2.6 ± 0.9 vs. 5.5 ± 1.0, *P *< .001). In the comfort group, 74 of 80 patients (92.5%) reported VAS scores ≤3 at 24 hours, compared with 12 of 80 patients (15.0%) in the conventional group (*P *< .001). No patient in either group required opioid analgesia; 6 patients in the conventional group took ibuprofen 400 mg as prescribed for VAS ≥4, vs. none in the comfort group.

### Anxiety Assessment

Pre-procedural SAS scores were lower in the comfort group (30.2 ± 5.7 vs. 46.2 ± 7.0, *P *< .001). Based on established SAS thresholds, 76 patients (95.0%) in the comfort group fell within the normal range (<45), compared with 30 patients (37.5%) in the conventional group. Post-procedural SAS scores at 24 hours remained lower in the comfort group (28.4 ± 4.7 vs. 42.0 ± 6.8, *P* < .001). The mean change in SAS from pre- to post-procedure was −1.8 points in the comfort group and −4.2 points in the conventional group, indicating that anxiety levels remained stable and low throughout the peri-procedural period in the comfort-oriented arm.

### Procedural Efficiency

Stent removal time, measured from cystoscope insertion to complete stent withdrawal, was shorter in the comfort group (1.90 ± 0.35 min vs. 2.20 ± 0.50 min, *P *= .014). No procedure required abandonment or conversion to alternative anesthesia in either group. Mean time from patient arrival to discharge from the day-surgery unit was not significantly different (88 ± 12 min vs. 92 ± 15 min, *P* = .08), as the additional time for preoperative education in the comfort group was offset by smoother intraoperative progress.

### Patient Satisfaction

Satisfaction scores were higher in the comfort group (4.6 ± 0.5 vs. 3.6 ± 0.7, *P *< .001). When satisfaction was dichotomized at the prespecified threshold (score ≥4 indicating satisfaction), 77 of 80 patients (96.2%) in the comfort group met this criterion, compared with 56 of 80 patients (70.0%) in the conventional group (*P *< .01). During follow-up telephone calls, patients in the comfort group frequently mentioned reduced procedural fear and appreciation for clear explanations, whereas conventional group patients more often reported unexpected discomfort.

### Complications

Within 24 hours following stent removal, complications were infrequent and mild in both groups (Table 2). Two patients (2.5%) in each group experienced transient macroscopic hematuria lasting less than 2 hours, which resolved spontaneously. All events were classified as Clavien–Dindo Grade I. No patient developed UTI, required emergency department evaluation, or was readmitted within 24 hours. The difference in complication rates was not statistically significant (*P* > .05).

## Discussion

Double-J stent removal is routinely performed in urology departments worldwide, yet many patients experience significant discomfort during and after the procedure. Traditional approaches prioritize technical efficiency but often overlook patient comfort and psychological preparation. Studies have consistently documented that 50%-60% of patients report moderate to severe pain during conventional cystoscopic stent removal, with anxiety levels frequently elevated due to uncertainty about what to expect.^4^^,^^17^ These experiences can reduce patient satisfaction, compromise compliance with follow-up care, and delay return to normal activities.

Various interventions have been proposed to mitigate procedural discomfort. Nonpharmacological approaches such as music therapy have shown modest benefits. Ölçücü et al. demonstrated that listening to binaural beats^18^ or classical music during cystoscopy and stent removal reduced anxiety and pain scores compared with controls.^19^ While these distraction techniques are simple and cost-effective, their effect sizes are generally modest, and they may be insufficient for patients with high baseline anxiety or low pain thresholds. Advances in stent design have also been explored. Magnetic ureteral stents, which can be retrieved without cystoscopy, have shown promise in reducing pain and procedure time.^20^ However, they are more expensive, not universally available, and carry a 5%-8% risk of premature dislodgement.^21^ Similarly, pull-string stents allow removal without instrumentation but are associated with inadvertent displacement.^22^ These design-based solutions address intraoperative discomfort but do not manage preoperative anxiety or postoperative symptoms.

Most existing interventions target isolated aspects of patient discomfort—either pain, anxiety, or procedural technique—and are applied at a single time point. Few studies have evaluated comprehensive, workflow-integrated models that address the entire patient journey. Qualitative research has revealed that patients undergoing stent removal commonly report anticipatory anxiety, concerns about pain and exposure, and symptoms persisting beyond 24 hours.^23^ These findings underscore that effective patient-centered care requires more than analgesia alone; it demands psychological preparation, procedural transparency, and structured follow-up. Recognizing these gaps, a comfort-oriented management protocol was developed, integrating preoperative education with relaxation techniques, intraoperative sedation combined with local anesthesia, pressurized irrigation-assisted stent extraction, and postoperative telephone follow-up.^24^ This approach addresses both physiological and psychological dimensions of discomfort throughout the peri-procedural period.

The results demonstrate that the comfort-oriented model reduced pain and anxiety within the 24-hour observation period. The VAS scores were lower in the comfort group at both immediate post-procedure (1.8 ± 0.8 vs. 4.2 ± 1.1) and 24-hour assessments (2.6 ± 0.9 vs. 5.5 ± 1.0), representing reductions of approximately 57% and 53%, respectively. Similarly, pre-procedural SAS scores were lower in the comfort group (30.2 ± 5.7 vs. 46.2 ± 7.0), with 95% of patients scoring in the normal range compared with 37.5% in the conventional group. These differences persisted at 24 hours post-procedure, indicating sustained benefit within the early post-procedural period.

The mechanisms underlying these improvements likely involve synergistic effects across multiple levels. At the psychological level, structured preoperative education establishes realistic expectations and reduces uncertainty, which are known to modulate pain perception through descending inhibitory pathways. Patients who understand the sequence of events and expected sensations report lower anxiety and pain, even when analgesic interventions are identical. The addition of deep-breathing exercises and progressive muscle relaxation further enhances autonomic regulation, reducing sympathetic arousal that amplifies subjective discomfort. At the pharmacological level, combining intravenous propofol with posterior urethral lidocaine infiltration provides both systemic anxiolysis and targeted peripheral analgesia. Propofol was selected for this protocol given its rapid onset, short duration of action, and predictable recovery profile, which are particularly well suited to outpatient day-surgery settings where turnover efficiency is a priority. Its use at sedative rather than anesthetic doses (OAA/S ≤3) avoids the airway management requirements associated with general anesthesia, and continuous monitoring of SpO_2_, blood pressure, and ECG ensured patient safety throughout. No procedure required conversion to general anesthesia, and no patient experienced sedation-related adverse events, supporting the feasibility and safety of this approach in a monitored outpatient cystoscopy environment. Urethral lidocaine blocks nociceptive transmission from instrumentation, complementing the systemic effect of propofol. At the mechanical level, pressurized saline irrigation facilitates urethral dilation and reduces friction between the stent and urethral wall, minimizing mucosal trauma during stent extraction and decreasing both intraoperative pain and postoperative dysuria. Taken together, these findings are consistent with recently reported data: Abdellatif et al^7^ demonstrated that pharmacological modulation of stent-related discomfort can yield clinically meaningful symptom reduction, and Wanifuchi et al^19^ confirmed that even simple non-pharmacological distraction during flexible cystoscopy produces measurable reductions in anxiety and pain. The results extend these findings by demonstrating that a comprehensive, multimodal approach addressing preoperative, intraoperative, and postoperative dimensions simultaneously produces larger and more consistent improvements than any single intervention alone.

However, a critical consideration is that the multicomponent intervention limits the ability to attribute benefits to specific individual elements. The large pre-procedural anxiety difference (30.2 vs. 46.2) suggests that preoperative education may account for substantial anxiety reduction, while immediate post-procedure VAS reduction (1.8 vs. 4.2) likely reflects direct analgesic effects of sedation and lidocaine. For resource-constrained settings, preoperative education with relaxation training may offer the most cost-effective first-tier intervention, requiring minimal resources yet producing significant anxiety reduction. Intravenous sedation with local anesthesia would constitute a second-tier intervention for patients with high baseline anxiety or low pain thresholds. Future factorial or component-dismantling studies are needed to quantify the independent contribution of each element and enable evidence-based tailoring to different clinical contexts.

Procedural efficiency was also improved, with stent removal time reduced by 14% in the comfort group (1.90 vs. 2.20 minutes, *P *= .014). Although this appears modest in absolute terms, it reflects smoother intraoperative progress due to better patient cooperation and reduced interruptions. The 30 minutes allocated for preoperative education was offset by shorter procedure time, reduced recovery room time due to fewer patient complaints, and smoother patient flow. Total outpatient time showed no significant difference (88 vs. 92 minutes, *P *= .08), indicating that the comfort-oriented model delivered substantial improvements in patient experience without compromising overall workflow. In high-volume day-surgery settings, these cumulative efficiencies can meaningfully improve resource utilization. Patient satisfaction was substantially higher in the comfort group, with 96.2% reporting satisfaction compared with 70.0% in the conventional group. During follow-up calls, comfort group patients frequently expressed gratitude for clear communication and reduced fear, whereas conventional group patients more often described unexpected discomfort. Safety was comparable between groups, with low complication rates (2.5% in each group) and no serious adverse events. The use of intravenous sedation did not increase the incidence of hematuria, UTI, or emergency visits. These findings support the feasibility and safety of incorporating sedation and advanced analgesic techniques into outpatient stent removal protocols. From a clinical perspective, the comfort-oriented model offers several pragmatic advantages.^25^ The protocol is straightforward to implement, requiring only standardized educational materials, existing anesthesia resources, and structured follow-up checklists. While intravenous sedation necessitates monitoring and trained personnel, the incremental cost is modest and likely offset by improved throughput and enhanced patient satisfaction. The findings have broader implications beyond stent removal; similar comfort-oriented protocols could be applied to diagnostic cystoscopy, URS, prostate biopsy, and other outpatient urological procedures.

This study has several important limitations. As a single-center investigation, generalizability may be limited by local practice patterns, patient demographics, and cultural factors. The absence of blinding for patients and operators introduces potential performance and detection bias, particularly for subjective outcomes such as VAS, SAS, and satisfaction scores. Patients aware of receiving “enhanced” care may have experienced expectancy effects that amplified perceived benefits, while conventional group patients may have reported more negative experiences. Future multicenter trials employing blinded outcome assessors and sham education interventions would strengthen causal inference. Incorporating objective physiological measures—such as heart rate variability, salivary cortisol, or skin conductance—would provide convergent validity for anxiety reduction claims independent of self-report. The patient satisfaction scale used in this study was developed specifically for this investigation and has not undergone formal psychometric validation; cross-study comparisons of satisfaction scores should therefore be interpreted with caution, and future research should employ externally validated patient-reported outcome instruments.

The 24-hour follow-up period represents a significant limitation. Delayed complications such as late-onset UTIs, persistent dysuria beyond 24 hours, or long-term impacts on patient willingness to undergo future urological procedures could not be assessed. Therefore, the conclusions are confined to the immediate peri-procedural experience and should not be extrapolated to long-term outcomes. Extended follow-up at 1 week, 1 month, and 3 months would be necessary to evaluate the durability of benefits and incidence of delayed complications. Sample size calculations were performed for primary pain outcomes but not for secondary endpoints, and smaller secondary effects should be interpreted cautiously. Cost-effectiveness analysis was not performed; formal economic evaluation would be necessary before recommending widespread adoption in health systems with limited budgets. Future research should focus on multicenter validation across diverse settings, mechanistic studies using objective biomarkers, component-dismantling trials to determine independent effects of each intervention element, long-term follow-up to assess delayed complications and chronic symptoms, and integration with digital health platforms for scalable delivery of education and remote monitoring. Extension of the comfort-oriented framework to other minimally invasive urological procedures could standardize patient-centered care across the specialty.^26^

In conclusion, this randomized controlled trial demonstrates that a comfort-oriented management model for daytime DJ stent removal reduces procedural pain and anxiety within the 24-hour peri-procedural period, enhances patient satisfaction, and improves procedural efficiency without increasing complications. By addressing the full spectrum of patient experience—from preoperative preparation through postoperative support—this approach provides a practical framework for implementing patient-centered care in outpatient urology settings. The model is safe, feasible, and warrants broader evaluation in multicenter trials with extended follow-up and component-specific analyses.

The comfort-oriented daytime DJ stent removal model is safe, feasible, and effective in reducing procedural pain and anxiety, shortening procedure time, and improving patient satisfaction. Its implementation in daytime urological surgery represents a practical approach to optimizing patient experience and clinical efficiency.

## Figures and Tables

**Figure 2.**
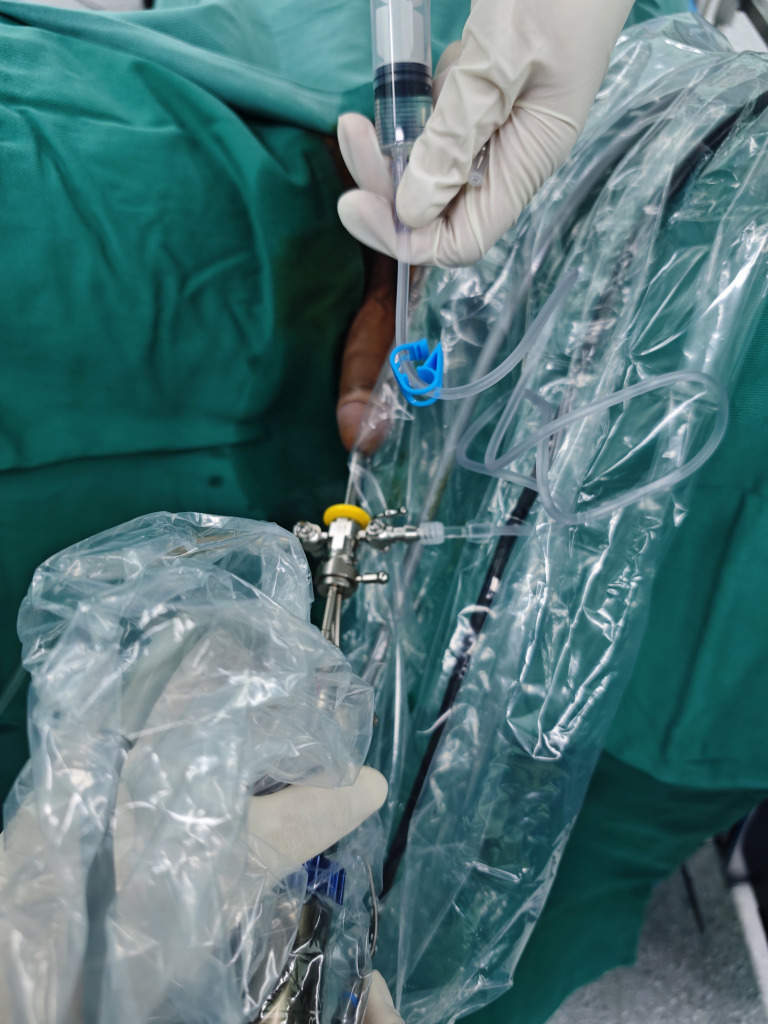
Procedural steps of comfort-oriented daytime Double-J stent removal (assistant-assisted slow injection of 10 mL of 1% lidocaine into the posterior urethra through the cystoscope-mounted irrigation channel, aiming to reduce urethral discomfort and early postoperative dysuria).

**Table 1.** Baseline Characteristics of Patients in the Comfort and Conventional Groups

**Characteristics**	**Comfort Group**	**Conventional Group**	***P***
Age (years)	45.0	46.4	.462
Sex (M/F)	48/32	46/34	.872
BMI (kg/ms)	24.3	24.7	.52
Surgery type (URS/PCNL)	68/12	71/9	.64
Stent duration (days)	10.4	10.1	.424
Prior UTI, n (%)	14 (17.5)	9 (11.2)	.367

BMI, body mass index; UTI, urinary tract infection.

**Table 2.** Comparison of Pain, Anxiety, Procedural Efficiency, Satisfaction, and Complications Between the 2 Groups

**Outcome**	**Comfort Group**	**Conventional Group**	***P***
VAS pain score	2.6 pain	5.5 pain	<.001
SAS (pre)	30.2	46.2	<.001
SAS (post)	28.4	42.0	<.001
Removal time (min)	1.90	2.20	.014
Satisfaction score	4.6	3.6	<.001
Satisfaction rate (%)	96.20	70.00	<.001
Complication rate (%)	2.50	2.50	1

SAS, Self-Rating Anxiety Scale; VAS, Visual Analog Scale.
